# Current perspectives on biosimilars

**DOI:** 10.1007/s10295-019-02216-z

**Published:** 2019-07-17

**Authors:** Frank K. Agbogbo, Dawn M. Ecker, Allison Farrand, Kevin Han, Antoine Khoury, Aaron Martin, Jesse McCool, Ulrike Rasche, Tiffany D. Rau, David Schmidt, Ma Sha, Nicholas Treuheit

**Affiliations:** 1 Cytovance Biologics, 800 Research Parkway, Suite 200, Oklahoma City, OK 73104 USA; 2 BioProcess Technology Group, BDO-USA Life Sciences Practice, 12 Gill St, Suite 5450, Woburn, MA 01801 USA; 3 Present Address: Joinn Biologics US, Inc, 2600 Hilltop Dr, Richmond, CA 94806 USA; 4 Project Farma, 810 W Randolph St, Chicago, IL 60607 USA; 5 Eppendorf Bioprocess Center, Rudolf-Schulten-Str. 5, 52428 Juelich, Germany; 6 616 Lagrange St, West Lafayette, IN 47906 USA; 7 Eppendorf Inc, 175 Freshwater Boulevard, Enfield, CT 06082-4444 USA; 8 Pfenex Inc, 10790 Roselle St, San Diego, CA 92121 USA

**Keywords:** Biosimilars, Quality, Analytical, Manufacturing, Mammalian, Microbial

## Abstract

In this work, an overview of the biosimilars market, pipeline and industry targets is discussed. Biosimilars typically have a shorter timeline for approval (8 years) compared to 12 years for innovator drugs and the development cost can be 10–20% of the innovator drug. The biosimilar pipeline is reviewed as well as the quality management system (QMS) that is needed to generate traceable, trackable data sets. One difference between developing a biosimilar compared to an originator is that a broader analytical foundation is required for biosimilars and advances made in developing analytical similarity to characterize these products are discussed. An example is presented on the decisions and considerations explored in the development of a biosimilar and includes identification of the best process parameters and methods based on cost, time, and titer. Finally factors to consider in the manufacture of a biosimilar and approaches used to achieve the target-directed development of a biosimilar are discussed.

## The diversity of biosimilars: an overview of the market, pipeline and targets **(Dawn M. Ecker, Tiffany D Rau)**

Since the approval of the first biopharmaceutical Humulin™ in 1982 [50], the biopharmaceutical industry has grown significantly with 2018 sales totaling $210B [6]. Within the past three decades, nearly 300 recombinant biopharmaceuticals have been patented and approved. With older biopharmaceuticals coming off patent, companies can now develop and sell copies of these off-patent biopharmaceuticals—commonly referred to as biosimilars. Although biopharmaceuticals are an established sector and available globally, biosimilars have only began to enter the mainstream of the market and this sector is in its infancy.

While the term biopharmaceutical is well understood within the industry, the term “biosimilar” is relatively new and there are often several terms improperly associated with biosimilars—including the words generic, biobetter and biosuperior. To appreciate why these terms do not apply to biosimilars, it is important to understand the definition of a biosimilar. Although the definition of “biosimilar” is not globally standardized between regulatory agencies, a comparison of how the regulatory agencies define the term biosimilar reveals significant resemblance between them:

- US FDA [53]: “A biosimilar is a biological product that is highly similar to and has no clinically meaningful differences from an existing FDA-approved reference product.”
- EU EMA [5]: “A biosimilar is a biological medicine highly similar to another already approved biological medicine.”
- WHO [31]: “Similar biotherapeutic product (SBP). A biotherapeutic product that is similar in terms of quality, safety and efficacy to an already licensed reference biotherapeutic product.”

The similarities among these regulatory agencies’ definition of a biosimilar can be distilled into two main themes:

- Biosimilars are not generics and are copies of already approved products that have proven to be similar to the original product (i.e., reference product) and
- Biosimilars must possess similarity in quality, safety and efficacy to an approved biologic product.

Although biosimilars and generics are both “versions” of brand name approved drugs, the terms biosimilar and generic are not interchangeable. The word “generic” is noticeably absent from the regulatory definitions listed above. Generics (chemically synthesized small molecule) are identical to the original product, while biosimilars (biologics synthesized by a living cell) are only highly similar to their original product. The lack of interchangeability between the use of the word generic and biosimilar directly relates to the terms “identical” and “highly similar”. As generics are chemically synthesized, the generic is chemically identical to the reference product. Biosimilars, however, are products which are made by a living cell and although two living cells may possess the same amino acid sequence for a protein, natural variations in glycosylation or protein folding may occur [56]. These cell-to-cell product differences are often referred to as “within-product variations” [55]. As biologics have very complex manufacturing paths, generating a biologic molecule that is identical to the reference product would be unachievable; however, generating a biologic molecule highly similar to the reference product is achievable, as the term “similarity” allows for the natural variability of the biologics to exist. Therefore, although the term generic is often used when discussing biosimilars, it is not applicable and incorrect. “Biobetter” or “biosuperior” is an additional term which has often been used when discussing biosimilars. Akin to the term generic, these terms are not applicable to biosimilars as the word “biosimilar” is a regulatory term which has a specified shortened pathway for approval [54]. The terms, biobetter or biosuperior, are not indicative of any regulatory pathway—but a marketing term for classification of new or second-generation products. Typically, these “biobetter” molecules may be similar to currently approved products, but the molecules are usually altered to improve attributes such as dosing regimen, safety, efficacy or immunogenicity [15]. Because of the significant differences in the molecular structure and molecule function, biobetters are considered new biological entities and would follow the standard approval pathway set by regulatory agencies, not a biosimilar pathway [15]. Examples of biopharmaceutical biobetters include:

- Neulasta^®^ (pegfilgrastim) which provides an improved half-life and dosing regimen when compared to Neupogen (filgrastim) [11],
- Kadcyla^®^ (trastuzumab emtansine): showed an improvement in standard of care for breast cancer when compared to Herceptin (trastuzumab) [21],
- Macrogenics Inc’s margetuximab: an (Fc-optimized) antibody with an optimized glycosylation profile to improve ADCC effector functions although the target is the same as Herceptin (trastuzumab) [42].

**Fig. 1:**
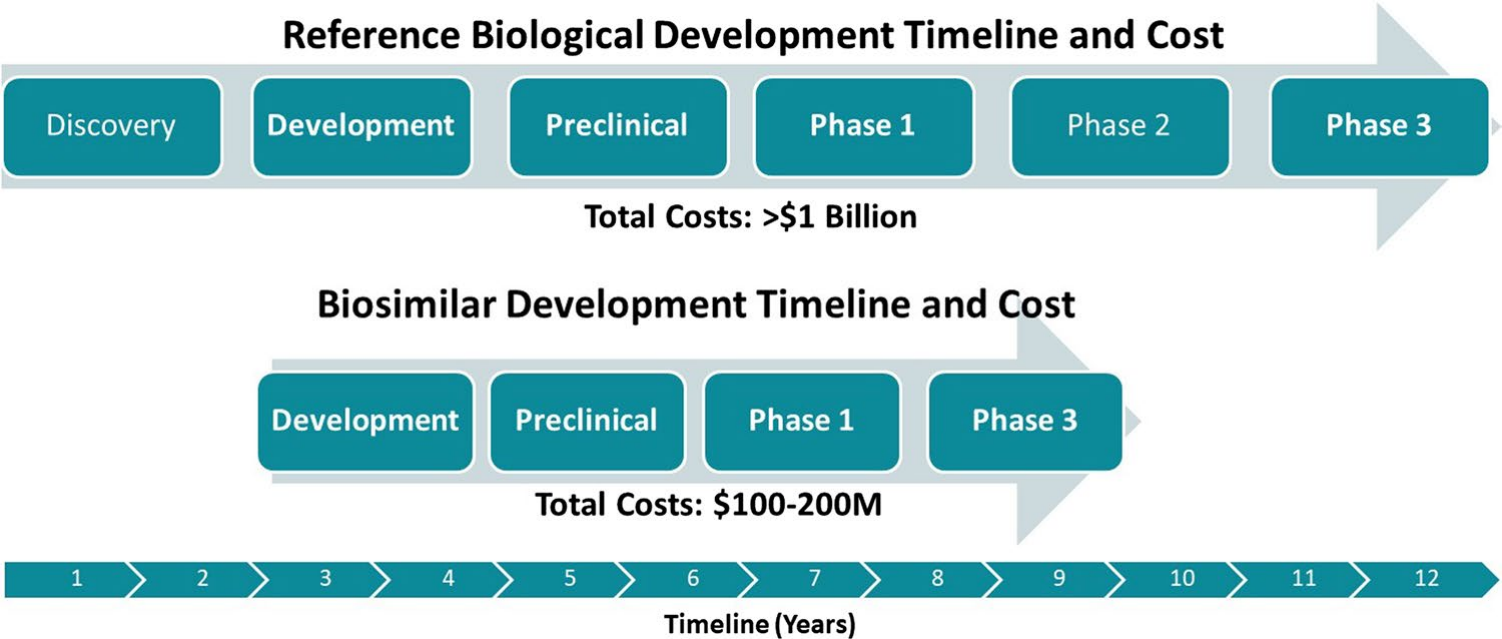
Originators versus biosimilars—development path [19]

As shown in Fig. 1, the biosimilar approval path differs from the standard approval pathway in time, which in turn affects cost. The typical standard biologics approval pathway from discovery to Phase 3 is approximately 12 years [19, 51]. Molecules following the standard biologics approval pathway submit an Investigational New Drug Application (IND) and enter Phase 1, to determine safety and dosage. If successful, the molecule will advance to Phase 2 where efficacy and side effects are determined and, if successful, will advance to Phase 3 to further study efficacy and monitor larger populations for adverse reactions. Biosimilar molecules, however, follow a shortened regulatory path [54]. As biosimilars are copies of marketed molecules with known product attributes, there is no discovery phase or initial efficacy required (i.e., Phase 2), thus shortening the path of development to 8 years (or less). Additionally, less work on development and the direct progression from Phase 1 to Phase 3 (as Phase 2 trials are not required) can translate to developing a biologic at 10–20% of the price of developing a new biological entity [16]. Biosimilar development emphasizes analytics as it must be proven that the molecule being developed as a biosimilar is “similar” to the originator molecule. On the other hand, the originator molecule manufacturer has the initial burden of ensuring that the product is safe and effective. Each product along with its respective indication(s) has a different clinical strategy. A minimum of two clinical studies are required for a biosimilar which would include one to compare pharmacokinetics of the originator product to the proposed biosimilar and then another clinical study to demonstrate clinical equivalence.

**Fig. 2:**
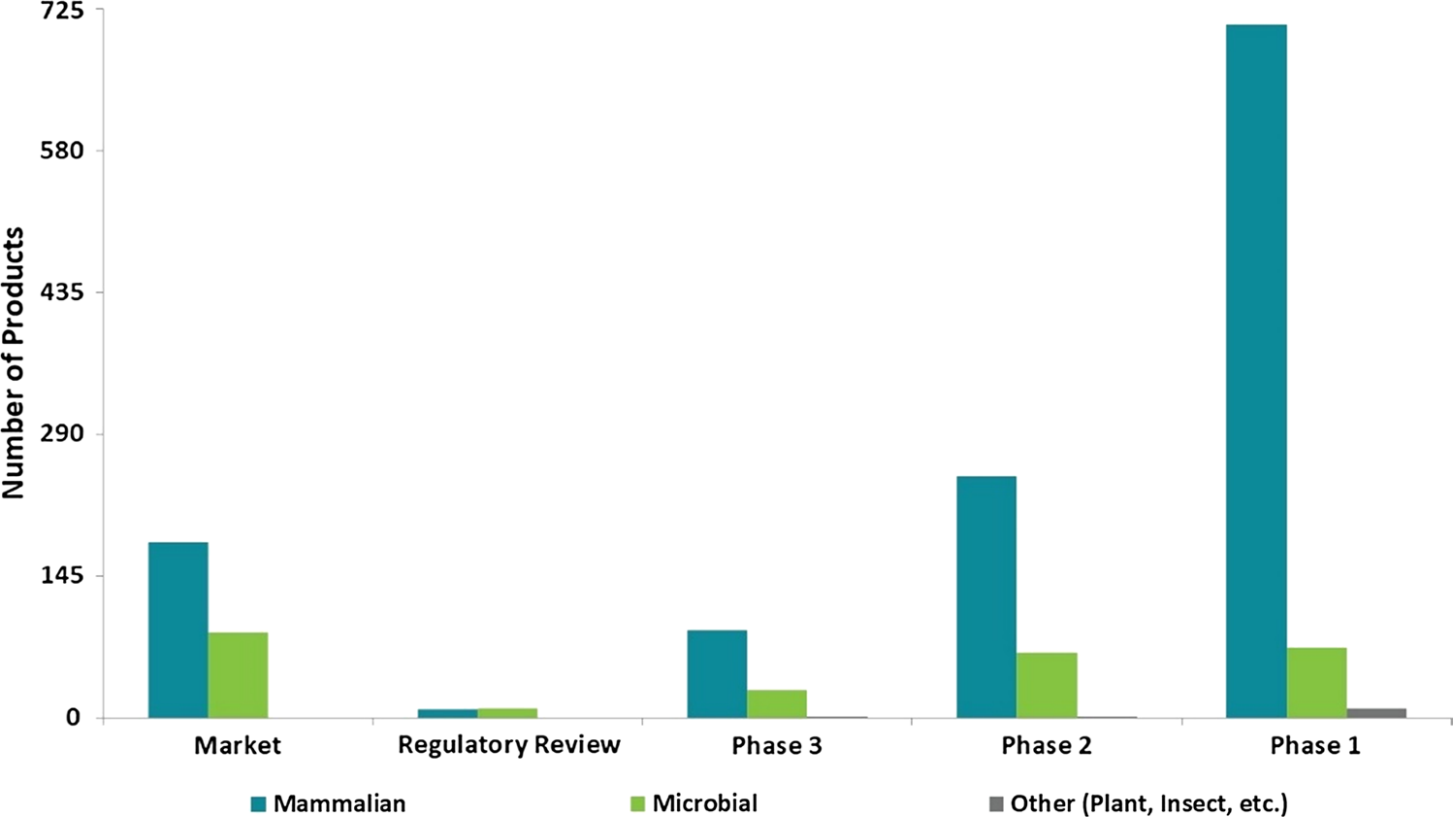
Current Biopharmaceutical Pipeline Distributed by Phase* and Production Technology. *Market refers to all US and EU Approved for Market Products, not all of which may be commercially available. The term microbial or mammalian refers to the type of host organism used to produce the biosimilar drug. *BLA* biologic license application, *MAA* marketing authorization application, *NDA* new drug application

**Fig. 3:**
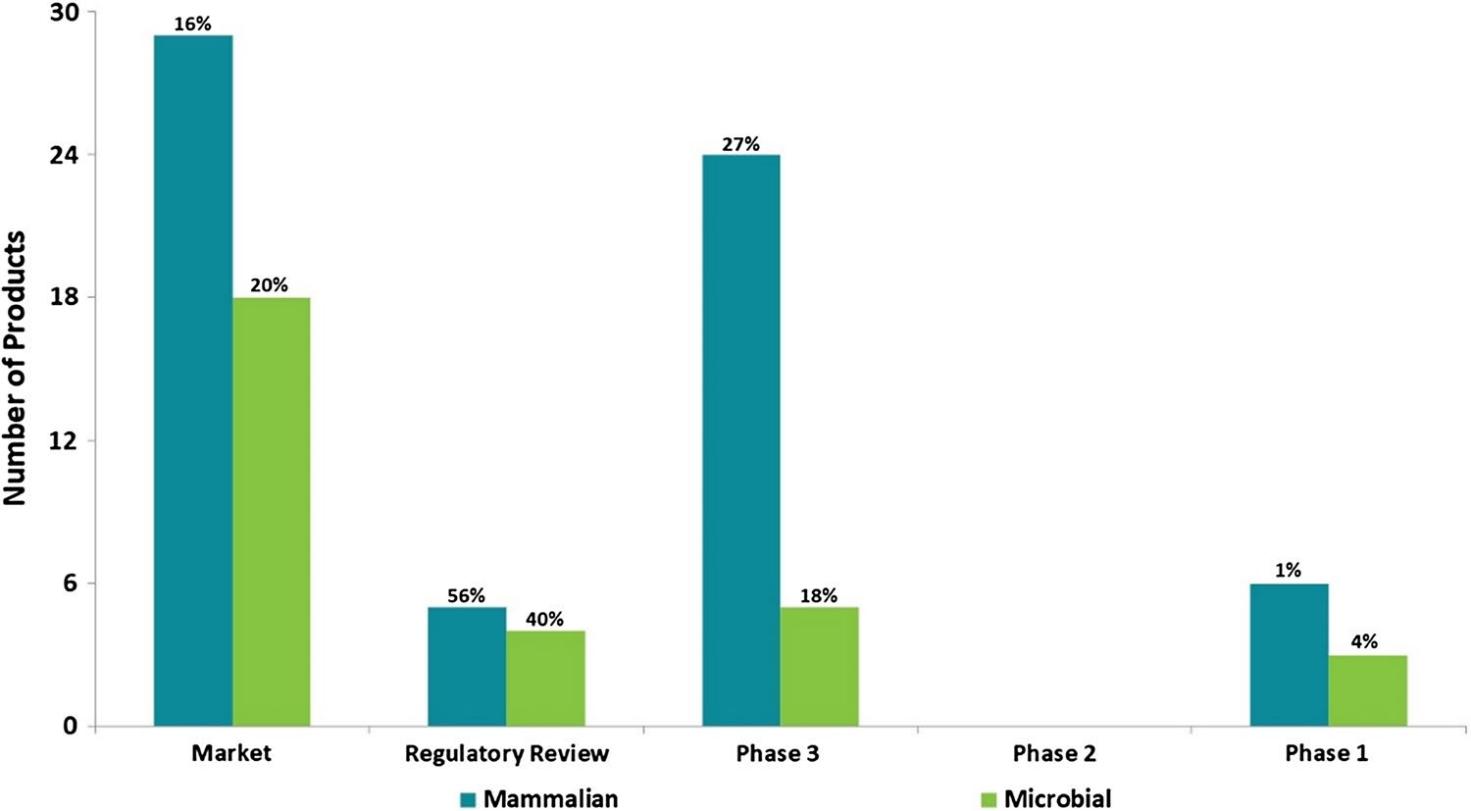
Current Biosimilar Pipeline Distributed by Phase* and Production Technology. *Market refers to all US and EU Approved for Market Products, not all of which may be commercially available. Percentages shown represent biosimilar share of biopharmaceuticals in a given Phase. Biosimilar products progress from Phase 1 directly to Phase 3. The term microbial or mammalian refers to the type of host organism used to produce the biosimilar drug. *BLA* biologic license application, *MAA* marketing authorization application, *NDA* new drug application

With the allure of biopharmaceutical market sales, decreased business risk and potential savings in development, it is not a surprise that companies are interested in developing biosimilars. Leveraging the information on recombinant biopharmaceutical products for the US and EU markets found in the bioTRAK^®^ database [6], profiles of the biopharmaceutical (Fig. 2) and biosimilar pipelines (Fig. 3) as of April 30 2019 are shown. While biosimilars are often discussed as a way to decrease healthcare costs [4, 40, 45, 62], they represent only small portion of marketed products and of the development pipeline.

Although the preceding figure indicates that nearly 50 biosimilar products have been approved for the market, not all approved biosimilars are available to patients. This delay in patient access is typically due to patent litigation, with each biosimilar company individually litigating against the originator. Regulatory bodies (i.e., FDA, EMA) which only provide market approval are not involved in understanding or heeding patent status. Two examples of this market access delay include adalimumab and teriparatide. Currently, there are six approved biosimilars to Humira; however, these products are available to patients in the EU as of October 2018 and will be available to patients in the US in January of 2023 [48]:

- Amgen: approved in 2016 for the US and in 2017 for the EU,
- Boehringer Ingelheim: approved in US and EU in 2017,
- Fresenius SE & Co KGaA approved in EU in 2019,
- Mylan and Fujifilm Kyowa Kirin Biologics: approved in the EU in 2018,
- Novartis (Sandoz): approved in US and EU in 2018,
- Samsung Bioepis, Biogen & Merck & Co: approved in EU in 2017.

Similarly, Gedeon Richter and Stada Arzneimittel have received EU regulatory approval for teriparatide biosimilars in 2017. However, these biosimilar products will likely not be available to European patients until sometime after 2019 when Forsteo’s patent expires [20].

**Fig. 4:**
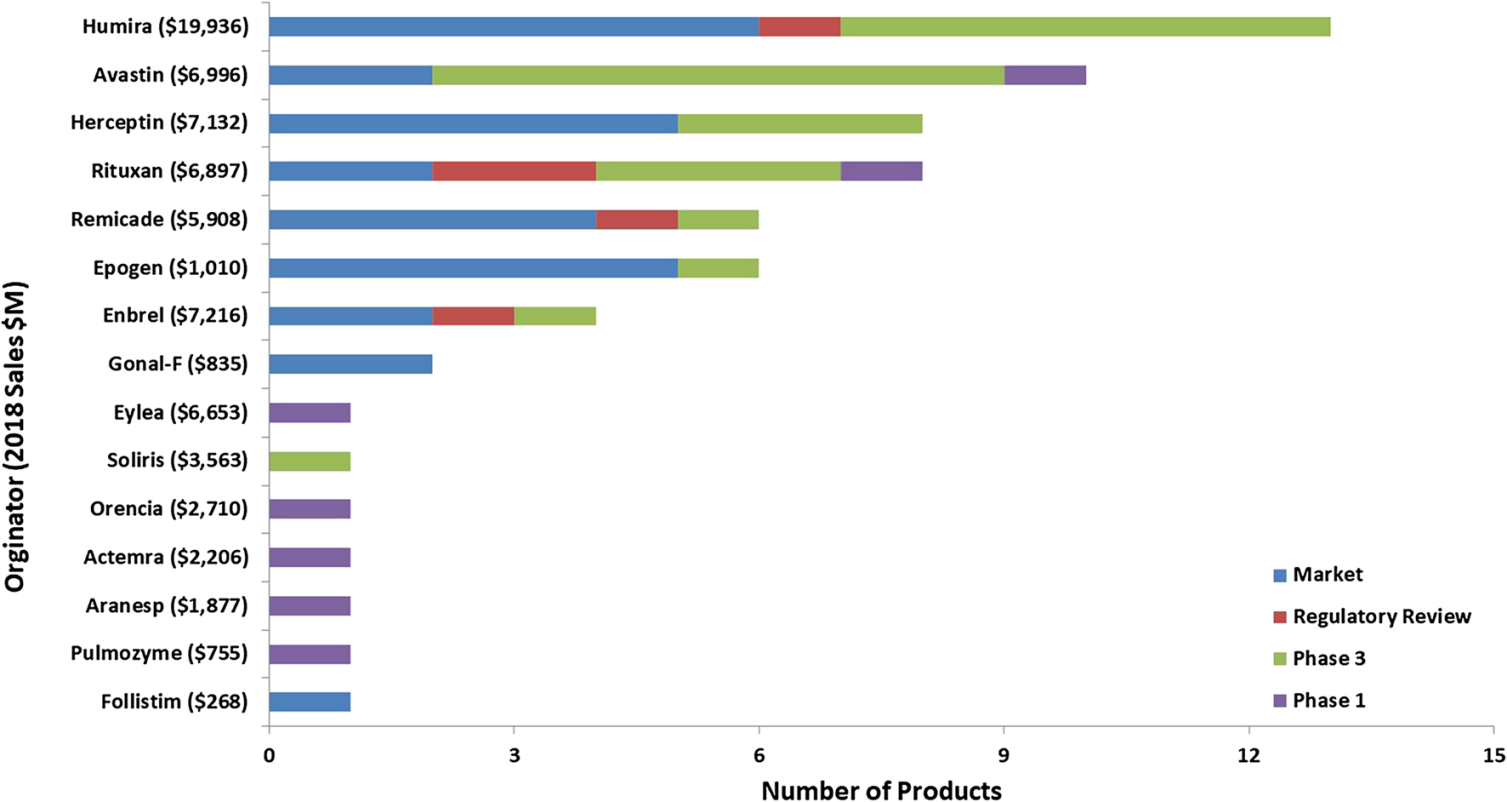
Mammalian-based biosimilar targets. The term Mammalian refers to the type of host organism used to produce the biosimilar drug. *BLA* biologic license application, *MAA* marketing authorization application

Looking closer at the mammalian biosimilar pipeline, among the biosimilars being developed, there are 15 originator molecules which are being targeted (Fig. 4). These 15 originator molecules represent nearly $74B in sales and 35% of all biopharmaceutical sales in 2018 ($210B). With hopes of obtaining a portion of the $74B market, there are a total of 37 companies involved in the development and/or marketing of the nearly 65 mammalian-based biosimilars. Of these 37 companies, the majority (86%) do not have a marketed product targeted by a biosimilar and the majority of these companies (75%) can be considered to be primarily focused on developing biosimilars as their product portfolios consist of at least 50% biosimilar products.

**Fig. 5:**
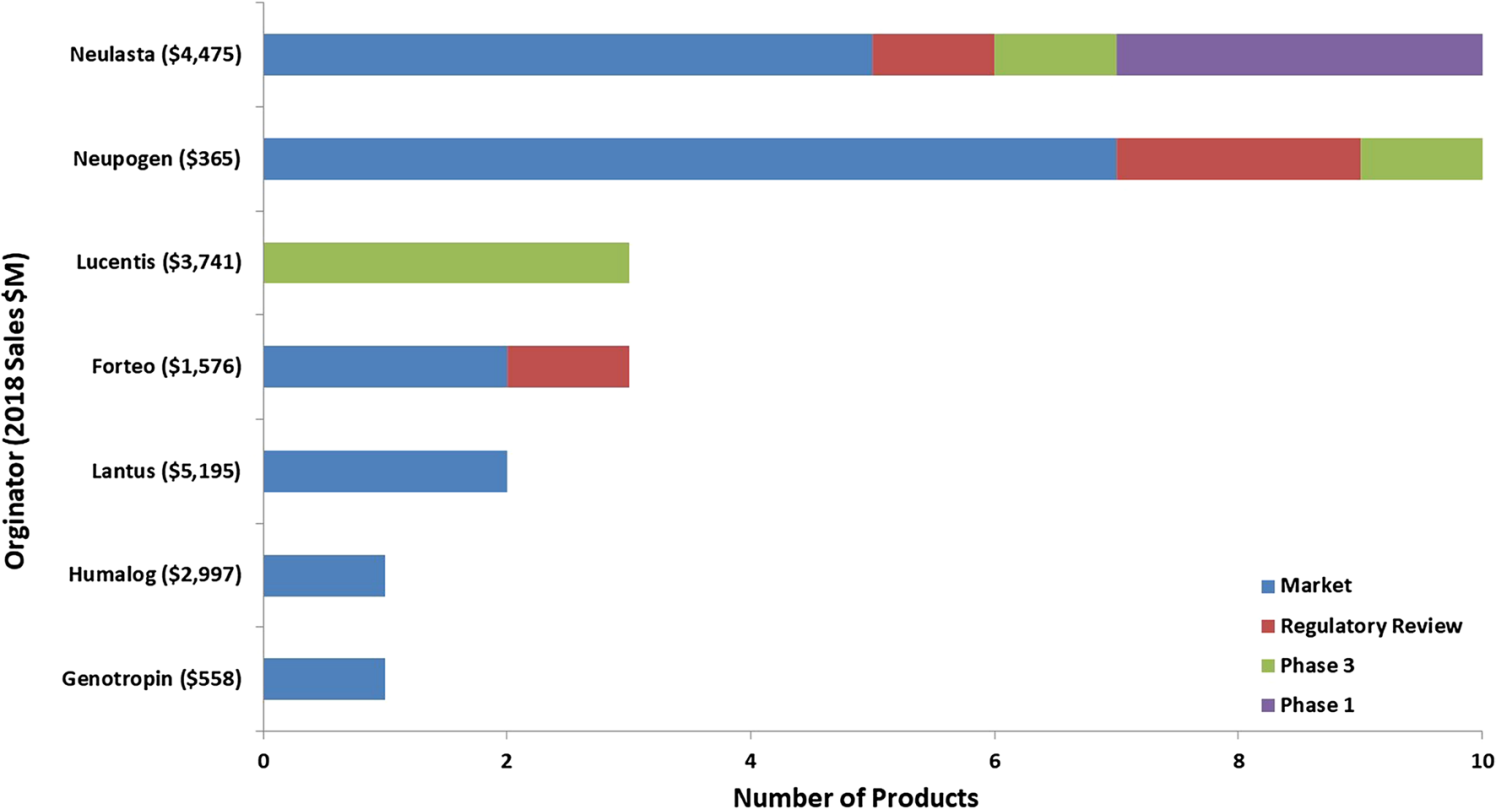
Biosimilar targets for microbial-based biopharmaceuticals. The term microbial refers to the type of host organism used to produce the biosimilar drug. *BLA* biologic license application, *MAA* marketing authorization application, *NDA* new drug application

For the microbial biosimilar pipeline (Fig. 5), there are only seven molecules currently being targeted by biosimilars, significantly fewer than the mammalian-based pipeline. These seven originator molecules represent $19B in sales and nearly 10% of all biopharmaceutical sales in 2018 ($210B). There are 26 companies involved in the development and/or marketing of the 30 microbial-based biosimilars. Of these 26 companies, the majority (81%) do not have a marketed product targeted by a biosimilar and the majority of these companies (86%) can be considered to be primarily focused on developing biosimilars as their product portfolios consist of at least 50% biosimilar products.

While biosimilars currently represent a small portion of the biopharmaceutical pipeline in the US and EU, currently marketed products will continue to lose patent protection. With the allure of a shortened regulatory timeline and significant savings in developing a biosimilar, it is likely that this market sector will continue to expand beyond the nearly 100 products and nearly 50 companies currently involved in biosimilars.

## Quality assurance in R&D for biosimilars **(Antoine Khoury)**

As discussed above, the pharmaceutical industry has seen substantial growth of biosimilars and an increase in the approvals of biologics [10]. With projected continuous growth, there is a definitive need for biosimilar R&D spaces to implement necessary quality management system (QMS) when applying for approval with the FDA. While biosimilars can rely on previously determined data for approval with the FDA, the approval process is no less rigorous. Chemistry, manufacturing, and controls (CMC) issues are often cited in FDA complete response letters (CRL). [59]. Therefore, how sponsors proactively address and improve the approach to CMC can enable a more rapid and robust timeline to the Biologics License Application (BLA) leading the way to product approval.

Per the FDA, a biosimilar manufacturer may partially rely on previous FDA determinations of a reference product by showing that the proposed biosimilar product is highly similar and has no clinically meaningful differences [54]. Quality data of the reference product must be used for this determination and, without a quality system for maintaining data, biosimilar manufacturers may find themselves generating full profiles of clinical and nonclinical data for the proposed biosimilar. By biosimilar manufacturers designing and implementing a QMS, the biosimilar industry can improve time to market.

While full traditional quality systems and 21 CFR Part 11 compliance and conformity to ICH Guidelines may restrict the flexibility that comes with R&D, there is a balance to be struck to ensure a structure is in place for tracking various systems while fitting the needs of R&D. Use of a balanced QMS can reduce the cost of development and curb regulatory barriers to innovation. Development and implementation of a QMS for an R&D environment will protect data through archivable, retrievable, and auditable systems. QMS also shows control over the equipment, process development, and changes to the process. A strong QMS will provide control for documents, learning, equipment, and change management. An integral part of a QMS may include a Document Management System, Learning Management System, Equipment Asset Management, and Change Management Process.

A document management system (DMS), will manage, track, and store documents, reviews, and approvals. Documentation may contain, but is not limited to, policies, plans, protocols, procedures, specifications, and requirements. For usage and application in laboratory operations, a DMS will operate for review and approval of standardized analytical procedures, experimentation records, and ad hoc deviations within experimentation. Implementation of a DMS allows for structure without reducing flexibility of operations and provides information for analysts to carry out consistent approaches. An electronic DMS, such as ACE Document Management System [1], can notify reviewers and approvers when a document is due and can be configured to require quality assurance sign off. A controlled DMS allows auditors to follow the course of work done and maintain data integrity principles for 21 CFR Part 11 compliance [18] and follows ICH Guideline for Good Clinical Practice [27]. DMS can be implemented through use of forms and approved formats, audit trails, and traceable records (time, date, operator, equipment used). Use of a DMS during R&D for biosimilars will greatly reduce time to market.

A learning management system (LMS) administers, tracks, and reports on-the-job education and training initiatives to appropriate individuals. For laboratory applications, LMS can be used to capture routine work as written in standard operating procedures (SOP). An LMS such as compliance wire generates reports confirming that personnel are trained on standardized procedures for equipment and processes. They can also notify personnel when new training is due and allow for monitoring of training compliance. LMS creates a place for storage and managing of resumes, trainings (completed and uncompleted), qualifications, areas of expertise, and education. Information is easily retrievable, captures training accomplishments for personnel, and allows for continuous process and professional development while maintaining 21 CFR Part 11 compliance. An LMS is recommended as part of ICH Guidance for Industry Q10 Pharmaceutical Quality System [29].

An equipment asset management (EAM) system manages, tracks, and stores calibration and maintenance activities for equipment and instrumentation. This includes part replacement and spare parts, calibrations, and work orders. EAM for asset assignment and tracking for operations, financials, and quality is imperative for laboratory applications. Electronic EAM systems like QAD [43] allow for monitoring of upcoming maintenance required on systems and can contain organization for assets and spare parts. For R&D purposes, EAM can be implemented for managing analytical equipment calibration schedules, ensuring alignment with requirements, and allowing closer monitoring of performance. Implementation of an EAM system demonstrates traceability and accuracy of equipment. EAM establishes reliability for data produced by equipment while maintaining 21 CFR Part 11 compliance and ICH Q7 Good Manufacturing Practice [28]. EAM reduces time lost to out of tolerance equipment and not functioning analytical tools, allowing for more time spent on R&D.

A change management process (CMP) formalizes the process to document, assess, approve, implement, and verify changes to process or equipment. For Deviation Reports Template in laboratory applications, CMP documents change, justifies change with an identified root cause, and assesses impact of change. Some CMP can function through a DMS, such as ACE which allows for full configuration of a uniquely defined CMP program. CMP documents changes to the process with new equipment to analytical pipeline. Changes outside of equipment are captured within CMP as well, such as changes to methods, SOPs, data collection, and reporting structure. Use of a CMP is recommended in the ICH Q10 Pharmaceutical Quality System [30]. An implemented CMP demonstrates control over procedural modifications, a routine for “non-routine” work. This will ensure no unnecessary changes are made, while providing justification to changes that are made.

In addition to systems for document, learning, equipment, and change management, an integral part of QMS is the quality asset management process. A defined process for quality asset procurement ensures acquired equipment is reliable and the data generated are meaningful. Identification of good automated manufacturing practice (GAMP) classification for laboratory systems is an important first step in asset procurement. GAMP Classes 2 and 3 are most common for laboratory applications.

GAMP Class 2 is defined for instrument and controllers with configurable and non-configurable firmware [39]. Required engineering actions and asset maintenance management for Class 2 include assignment of asset identifier ID (make, model, cutsheets), and document configuration and versioning control. Instrument is to be entered into EAM for preventative maintenance, calibration, and inventory spares, while DMS captures engineering turnover packages (specifications, manuals, drawings, data sheets). No validation actions are required for GAMP Class 2 as the instrument is compliant per the required engineering actions and asset maintenance management.

GAMP Class 3 is defined for commercial off-the-shelf (COTS) equipment and packages with existing code and setpoints. Required engineering actions and asset maintenance management for Class 3 include assignment of asset identifier (same as for Class 2), and documentation of COTS system installation and operations. The system is required to be entered in an EAM system for asset assignment, tagging, labelling, calibration, PMs and inventory spares. Engineering turnover packages and parts lists are to be entered and maintained in DMS. Validation actions are required for GAMP Class 3 systems. For COTS systems, a series of commissioning and qualification documents are required deliverables. System sustainability is required to validate system integration into infrastructures and systems for 21 CFR Part 11, user logins, historian and trending data, retrievable and accessible data storage.

Validation activities for GAMP 3 COTS systems follow the traditional V-model. Prior to purchasing equipment, necessary specifications and requirements for the system are to be defined. A COTS commissioning plan and requirement (CPR) captures these requirements as well as the commissioning and qualification (C&Q) strategy. Once the equipment that aligns with CPR is procured and brought on site, a receipt verification (RV) is to be performed prior to installation. The RV verifies that the system is received undamaged, confirms the inclusion of equipment shipping documents, verifies the make and model, and records the turnover packages (if applicable). After a successful RV, the system is ready to be installed and for commissioning testing to begin. Testing for verification of system requirements per the CPR and functionality is completed through a COTS commissioning verification or qualification (CCV or CCQ). An installation or operational qualification (IQ or OQ) may be executed if applicable for additional testing of CPR. When testing is complete, commissioning activities are summarized, and testing traced per requirements in a COTS commissioning report (CCR).

Performance qualification (PQ), if applicable, is performed to ensure the system consistently meets functional requirements in various scenarios including worst case scenarios and system capability limits and system failure. PQ activities, results, and findings are summarized in the performance qualification summary report (PQSR). Documents used for commissioning and qualification are reviewed, approved, and managed by appropriate parties within the DMS. Commissioned systems will require routine maintenance that can be tracked and managed through EAM. Routine maintenance such as calibration, part replacements, or deep cleanings can all be tracked through the appropriate channels within the EAM. For changes to equipment or processes outside of the scope of commissioning and maintenance procedures, CMP is used.

Implementation of quality systems for documentation, learning, equipment, and change management benefits R&D spaces by providing systems for traceable, trackable data. Use of QMS and defined asset procurement process can greatly reduce the time for process development and application for biosimilar approval. Having QMS to show reliable lab systems and storage for archivable, retrievable, and auditable data allows for efficient process development within biosimilar R&D spaces.

## Advances in analytical similarity **(Nicholas Treuheit)**

**Fig. 6:**
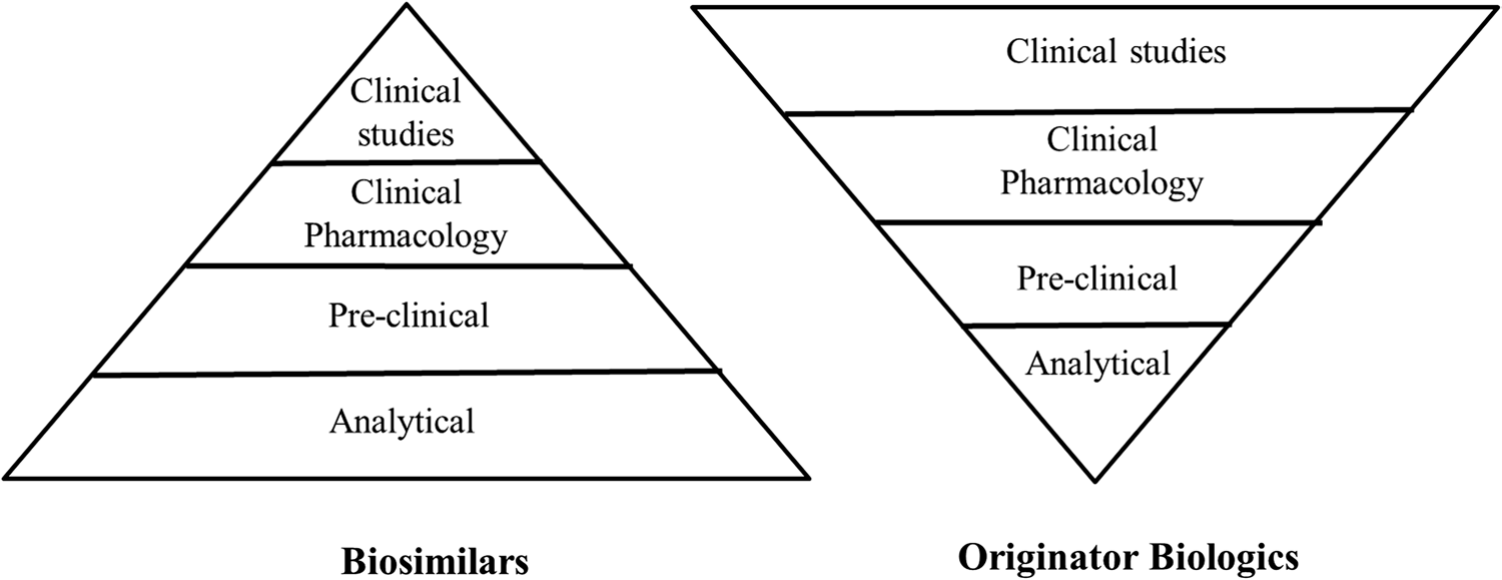
The canonical diagram for drug development for biosimilars compared to originator biologics

The canonical diagram for drug development is an inverted pyramid built upon a narrow foundation of analytical biochemistry that expands dramatically with the transition into pre-clinical and clinical testing [16]. Intuitively, this makes sense because of the stringent testing required by the FDA to demonstrate safety and efficacy. However, with the adoption of biosimilars both in the US and internationally, the classic dogma has been inverted (Fig. 6). Because safety and efficacy of the original drug have already been well established by the innovator, the base for biosimilar development becomes the extensive analytical characterization coupled with process development required to establish sufficient bio-similarity to the originator biologic [38]. With ample comparability testing, a biosimilar candidate can justify far less extensive clinical testing [60] and, as such, the development costs are significantly less than those required for an original drug.

Despite the importance of analytical characterization in biosimilar development, currently there is limited FDA guidance on the specific approach to demonstrating similarity [25]. Instead, there is a generic framework based around a “totality of the evidence approach.” Thus, the burden of designing a sufficiently rigorous analytical characterization package falls to biosimilar developers, which presents the challenge of being as thorough and expansive as possible. A number of biosimilars which have been approved in the US, such as Zarxio^®^ [24] and Fulphila™ [26], have helped establish a framework for creating a robust package of analytical similarity data. However, this process is continually being refined as technology advances and new or supplementary approaches to analytical characterization become available. Dialogue with the FDA throughout the development process is very important for the product being developed by the biosimilar developer.

Pfenex has based its approach to biosimilar characterization on published work [7, 22], review of other biosimilar FDA submissions [24, 26], and its extensive in-house expertise in analytical characterization of proteins produced by the *P. fluorescens* expression platform. Two characterization areas outlined by the FDA are structural and functional analyses, but the specific characterization or type of analysis is somewhat open to interpretation. It is clear that structural analysis should focus on primary, secondary, tertiary, and quaternary elements of the protein of interest. Structural analysis should also include characterization of any degradation products, intentional modifications, and post-translational modifications, including glycosylation which can pose a particularly difficult challenge in demonstrating similarity. There are a number of ways to characterize modifications in protein biologics including electrophoresis (both gel- and capillary), liquid chromatography (including reversed-phase, size exclusion, and ion exchange), differential scanning calorimetry, and mass spectrometry. In particular, liquid chromatography is the backbone of purity characterization and it is crucial for analyzing the sugar content in glycosylated biomolecules.

The structural elements can be analyzed using a systematic approach. Primary sequence can be confirmed using DNA sequencing and protein mass spectrometry, which typically uses highly specific proteases to generate peptides that can be used to confirm the amino acid sequence of the protein of interest [57]. Secondary structure can be identified using a variety of techniques such as Circular Dichroism, which can be used to characterize alpha helix, beta sheet, and random coil structural content [44]. IR spectroscopy can also be used to probe changes in amide backbone secondary structure and side chain chemistry [64]. Higher order tertiary and quaternary structure require different sets of experiments such as fluorescence spectroscopy [33], NMR [3], hydrogen–deuterium exchange mass spectrometry (HDX-MS) [23], and X-ray crystallography. Each of these techniques can yield detailed structural information and offer a strong base for making similarity conclusions.

In addition to structural characterization, functional characterization using in vivo and/or in vitro studies to confirm that biosimilar pharmacologic activity is similar to the reference must be performed. There are a variety of biological cell assays, immobilized binding assays, and substrate-binding kinetic assays that can serve as an excellent foundation. In vitro assays may include surface plasmon resonance, biolayer interferometry, isothermal titration calorimetry, plate-based assays, and others [12]. In vivo and biological assays may also be warranted, especially if different cell lines, formulations or other processes are different for the biosimilar. In vivo assays can help establish similarity in toxicity profile or highlight unexpected toxicity due to a different manufacturing impurity profile [32] and offer critical data to show real effects and interactions resulting from a drug of interest before beginning clinical testing. Finally, to build on these classical measurements, we believe a comparability package can be bolstered by also considering protein dynamics and attempting to connect the structural and functional analyses. To that end, we have been using hydrogen–deuterium exchange to probe biosimilar interactions more extensively, beyond just a protein’s isolated behavior in solution. This gives us a more thorough understanding of the structure–function relationship between a reference product or a biosimilar and their target. In turn, this should significantly improve the overall quality of analytical packages and strengthen similarity arguments in advance of clinical testing.

## Contract research and development perspective on biosimilar analytical development **(Allison Farrand, Aaron Martin, David Schmidt)**

As described in Sect. 1, biosimilars continue to gain more traction in the biopharmaceutical market. The FDA approved the first biosimilar in 2015 and since has approved a total of 16 biosimilars (three in 2016, five in 2017, and seven in 2018) [9, 49]. Concurrently, the relationship between pharmaceutical companies and contract development and manufacturing organizations (CDMOs) continues to evolve into a broad partnership model involving development, testing, production, and research [46]. The evolution of outsourcing development activities creates unique challenges for a CDMO working with pharmaceutical companies to develop biosimilar products. This section seeks to describe the perspective of a CDMO R&D department on the development of an analytical program for a biosimilar product, focusing on some of the challenges associated with establishing a biosimilar analytical development strategy as well as approaches to counteract these challenges and create a strong analytical program.

The first activity in a biosimilar project for a CDMO is preparation of a detailed contract. Prior to establishing a contract, adequate detail on the biosimilar project, requested activities, required timelines, and any historical data needs to be fully understood by the CDMO [14]. A typical CDMO business model includes predefined services with built-in assumptions on technical activities and resource allocation. This model allows for a structured statement or scope of work (SOW) with defined milestones to manage the execution, resource cost, and timelines of the services. For biosimilars, CDMOs should adopt more flexible scopes of work (SOWs), with freedom to make alterations in scope without changing contract details. Technical teams need the ability to divert resources with respect to progression of the project plan. Adherence to the traditional model with strict scopes would hinder the development process due to issuing and approving contract change orders. However, adequate scope boundaries are necessary to ensure costs are controlled. Thus, clear communication between the client and CDMO is critical to build SOWs that can meet the project requirements.

In addition to challenges associated with the preparation of a contract, additional challenges are faced related to the agreement of project timelines. Biosimilar projects have an abbreviated approval process and accelerated project timelines can be expected [52]. However, regulatory agencies view the analytical similarity data as the foundation of biosimilar development; therefore, appropriate timelines become a critical factor [37]. Additional attributes a CDMO should possess include experience for efficient development strategies for high-quality methods, prioritization of test method development for process development support, and adequate resources to maintain timeline projections. A critical aspect of method development that can impact project timelines is the parallel process development that requires analytical data to make informed decisions. This often leads to analytical program dependencies requiring operation-ready test methods. The order of execution as well as resources required for both analytical development and process development activities must be taken into careful consideration for a successful biosimilar development program. A CDMO company must be able to leverage experience and expertise to drive efficient and achievable project timelines.

After the contract and project timelines are agreed upon, the first analytical development stage of a biosimilar program is the initiation of test method development services. A prioritized method development plan matched with the CDMO resource availability and overall project plan is essential. The analytical techniques involved in determining the strength, identity, safety, and purity are typically developed first. Test methods developed here will be utilized for process development decisions, manufacturing release and stability testing by a quality control laboratory, and demonstration of reference product similarity. During the method development, the client should provide the CDMO with information regarding the potential critical quality attributes (CQAs), clinically non-relevant attributes, and supply representative material for development. After the method parameters are established, pre-validation, robustness, and stressed stability studies are required to guide optimization strategies. Analytical techniques that provide characterization data including primary structure, higher order structure, co- and post-translational modifications (PTMs), and additional functional assays are typically developed in parallel with a different set of resources. A clear strategy for the prioritization of test methods, resources, responsibilities of client, CDMOs, and any external vendors is essential for a successful test method development service.

The next stage in the biosimilar analytical development program involves comparing the biosimilar to the reference product, establishing the quality target product profile (QTPP), and setting manufacturing release and stability specifications. These studies are critical to biosimilar development since demonstration of similarity through thorough process and product characterization can significantly impact the need for clinical testing of the biosimilar [8]. For this stage multiple lots of reference product, often up to ten lots, and as many representative lots of biosimilar as available are analyzed by all release, stability, and characterization test methods [17]. It is important that forced degradation studies are performed to identify, quantitate, and compare product-related degradants. The client will have the responsibility to defend the degree of similarity to the regulatory authorities. If the biosimilar does not meet the client’s requirements for “highly similar,” further process development could be required. These comparability studies also generate data from multiple lots of the reference product and can establish the manufacturing variability of the reference product. Such data can be very informative and lead to meaningful manufacturing release specifications for the biosimilar. For a successful partnership on the preliminary comparability assessments, the client must drive the similarity study design and the CDMO must be flexible and sensitive to the client’s requirements.

Compared to new drug development, increased importance is placed on the early analytical data sets for a biosimilar due to the potential impact of decisions. It has become an expectation for quality to be integrated early into the biosimilar development program. Establishment of an R&D quality management system (R&D QMS) can help a CDMO meet these high-quality standards. The R&D QMS should be designed to have audit ready documentation without the full vigor of Quality Assurance (QA) oversight. Many of the development activities must operate with flexible study designs and thus cannot be subject to many of the QA programs designed for full, current GMP guidelines. The R&D QMS should, at a minimum, include equipment calibration, document control, and data integrity systems that apply to both new drug and biosimilar development.

Pharmaceutical companies continue to invest in biosimilar products and outsource manufacturing development projects to CDMOs. CDMO perspective when establishing an analytical development program from a biosimilar client and strategies to overcome challenges have been presented. Also, the importance of an early R&D QMS is presented. As more time and resources are applied to develop a process and characterize a biosimilar, a more flexible CDMO model with increased quality standards is developing [8]. Even with a flexible model, the success of the biosimilar analytical development program is attributed to the collection of experience, knowledge, and expertise gained through the partnership between biosimilar company and CDMO.

## Process development for a biosimilar product **(Kevin Han, Ulrike Rasche, Ma Sha)**

In the development of any biopharmaceutical process, including those involving biosimilars, decisions on the best process parameters and methods are made based on cost, time, quality and titer comparisons. The objective of this section is to compare the performance of batch, fed-batch, and perfusion processes—the three primary methods for production of a typical biosimilar. For this comparison, the cell growth, the metabolic profile, and antibody production were analyzed. Stirred-tank bioreactors at bench scale were used to perform the study. The purpose was to compare process performance in a comparably small volume to save resources, with the option to scale-up the process to larger volumes.

Human monoclonal antibodies (hmAbs) produced in CHO cells have played a major role in the diagnostic and therapeutic markets for decades. The number of antibodies gaining approval promises to increase in the future as many blockbuster mAb-based treatments reach the patent cliff. Indeed, process development and optimization are underway in many R&D facilities as companies race to develop biosimilars, or highly similar copies of currently approved therapeutic hmAbs. During upstream process development, the process mode is one of the factors to be considered. In a batch process, all nutrients are supplied in the initial base medium. In a fed-batch process, nutrients are supplied during cultivation. In perfusion processes, medium is circulated through a growing culture to allow waste removal and nutrient supply simultaneously.

A suspension CHO cell line from TPG Biologics, Inc., expressing hmAb was used as a potential biosimilar. All bioprocess runs were controlled with the same Eppendorf BioFlo 320 bioprocess control station. No hardware changes were necessary between the individual runs, aside from the vessel and the motor. The batch process was performed in a 2 L water-jacketed autoclavable glass vessel. For the fed-batch process, a BioBLU 5c Single-Use Vessel was used; the perfusion process was carried out in a BioBLU 5c Single-Use Vessel with a custom perfusion dip tube assembly. Both single-use vessels could serve working volumes from 1.25 to 3.75 L. Vessel bodies and head plates of the BioBLU Single-Use Vessels comprise single-layer injection-molded plastic. No additives such as softeners were used. Based on the standardized cell culture test developed by the DECHEMA working group, “Single-Use Technology in Biopharmaceutical Manufacturing” [13], the effects of leachables from the BioBLU 0.3c, 1c, 5c, and 14c single-use vessels on a CHO cell culture were tested. There was no observation of any negative effects caused by leachables from vessel material on growth, viability, and metabolic profile of the cell line tested.

Agitation, gassing, pH and DO control, and inoculation density were the same for each of the three experiments [61]. The cells were cultivated initially at 37 °C. In the batch run, the temperature was held constant. Since a temperature shift to 32 °C is a common practice for increasing CHO cell protein expression, the temperature was decreased in the course of the fed-batch and perfusion processes. In the fed-batch run, the temperature was decreased to 32 °C upon the initiation of feeding, and in the perfusion processes the temperature was shifted to 32 °C at day 7.

### Cell density and antibody titers in batch, fed-batch, perfusion processes

The batch process was run for 9 days. It resulted in a peak viable cell density of 1.5 × 10^7^ cells/mL on day 6, which then sharply declined. On day 5, the cells had consumed all of the initially supplied glucose. The lactate concentration increased up to 2.2 g/L at day 4. The lactate concentration decreased when time-shifted to the glucose consumption, which suggests that the cells underwent a metabolic switch from glucose to lactate consumption. The ammonia concentration continuously increased up to 9 mmol/L on day 9 as a result of cell death. Within 9 days, the culture produced 0.24 g hmAb/L for a total amount of 0.9 g hmAb from 3.75 L working volume.

The fed-batch process was run for 16 days. Starting from day 3, the culture was fed daily with CD EfficientFeed C AGT Nutrient Supplement and glucose. As a result, the glucose concentration stayed above 3 g/L throughout the entire process. A peak viable cell density of 2 x 10^7^ cells/mL was achieved. In contrast to the batch process, the viability stayed close to 100% even after 14 days of culture, most likely because of the constant supply of glucose and other nutrients. Feeding the culture also delayed the onset of lactate consumption that began around day 5 and was not depleted until day 10. Similarly, within the first 5 days of the process, the concentration of ammonia increased to approximately 4 mmol/L, then dropped back to initial levels temporarily before climbing to toxic levels late in the run. This phenomenon is attributed to metabolic changes that occur in response to high lactate concentrations, as has been documented previously [36]. Within 16 days, the culture produced 1.2 g of hmAb/L for a total amount of 4.5 g of hmAb from 3.75 L final volume.

For the perfusion process, a BioBLU Single-Use Vessels was connected with a Repligen ATF-2 alternating tangential flow filtration device to separate the cells from the media with continuous removal and replacement of cell culture fluid. The perfusion process was run for 14 days. The process reached a peak cell density of 7.4 × 10^7^ cells/mL, at over 90% viability, and produced 3.04 g of hmAb/L for a total antibody amount of 11.4 g of hmAb from 3.75 L final volume. In total, the fed-batch process produced fivefold more antibody than the batch process. The total antibody titer of the perfusion process was 12-fold higher compared to the batch process. Simple batch culture may be the least expensive and fastest approach to produce small quantities of hmAb. The fed-batch and perfusion process produced more antibody, but over a substantially longer period, and in case of the perfusion process using a much larger quantity of cell culture medium. The choice of which culture mode to use for production will depend on the quantity of hmAb required, the available time, and taking equipment and medium costs into account.

### Evaluation of bioprocess scalability

**Fig. 7:**
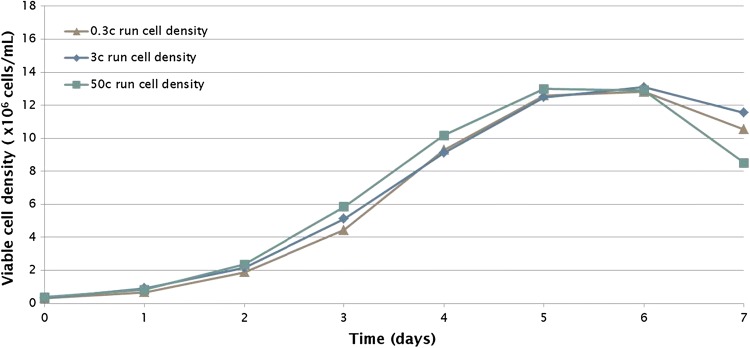
Cell growth profiles in a batch process. Viable cell densities were determined in cultures in the BioBLU 0.3c (0.25 L) Single-Use Vessel, the BioBLU 3c (3.75 L) Single-Use Vessel, and the BioBLU 50c (40 L) Single-Use Vessel. There were no replicates for these runs

**Fig. 8:**
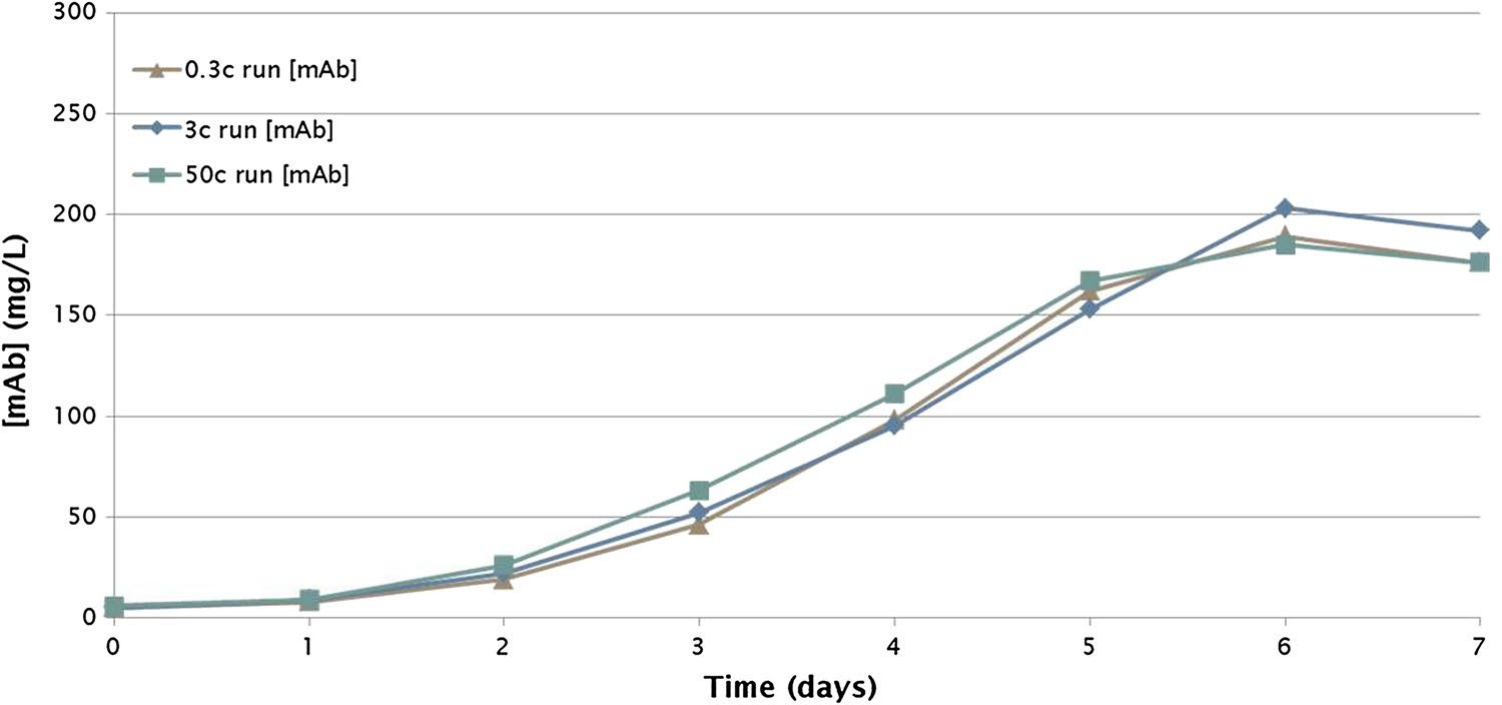
Antibody production in a batch process. mAb concentrations were determined in cultures in the BioBLU 0.3c (0.25 L) Single-Use Vessel, the BioBLU 3c (3.75 L) Single-Use Vessel, and the BioBLU 50c (40 L) Single-Use Vessel. There were no replicates for these runs

Different process modes such as batch, fed-batch and perfusion were evaluated at bench scale. As over the course of bioprocess development more material is needed for characterization and trial runs, the process scalability has to be kept in mind. The scale-up capabilities of Eppendorf BioBLU Single-Use Vessels for cell culture from small to bench scale to pilot scale were investigated for a batch process. Maintaining a constant power input/volume ratio (P/V) between vessels is one of the most prevalent strategies for scale-up. The mAb production process in CHO cell cultures was scaled up by keeping P/V constant across scales. BioBLU 0.3c Single-Use Vessels (maximum working volume of 0.25 L), BioBLU 3c Single-Use Vessels (maximum working volume of 3.75 L), and BioBLU 50c Single-Use Vessels (maximum working volume of 40 L) to represent an approximately tenfold scale-up between steps were used. The cell growth patterns (Fig. 7) and mAb production profiles (Fig. 8) were very similar at working volumes of 0.25 L, 3.75 L, and 40 L, indicating that keeping P/V constant led to suitable operating parameters for scale-up.

Decision on a process mode based on time, cost, and titer comparisons, and scale-up of the process from the research to the production stage are integral parts of biopharmaceutical upstream process development. In this section, the cell growth, metabolic profile, and antibody production in batch, fed-batch, and perfusion mode were compared using the same bioprocess control station and without making hardware changes between individual runs. Furthermore, the scale-up capabilities of the bioreactors used were demonstrated. This study gives an example of how flexible and scalable bioprocess equipment can help simplify bioprocess development and aid process transfer from development to manufacturing.

## Manufacturing of biosimilars **(Frank K. Agbogbo)**

**Fig. 9:**
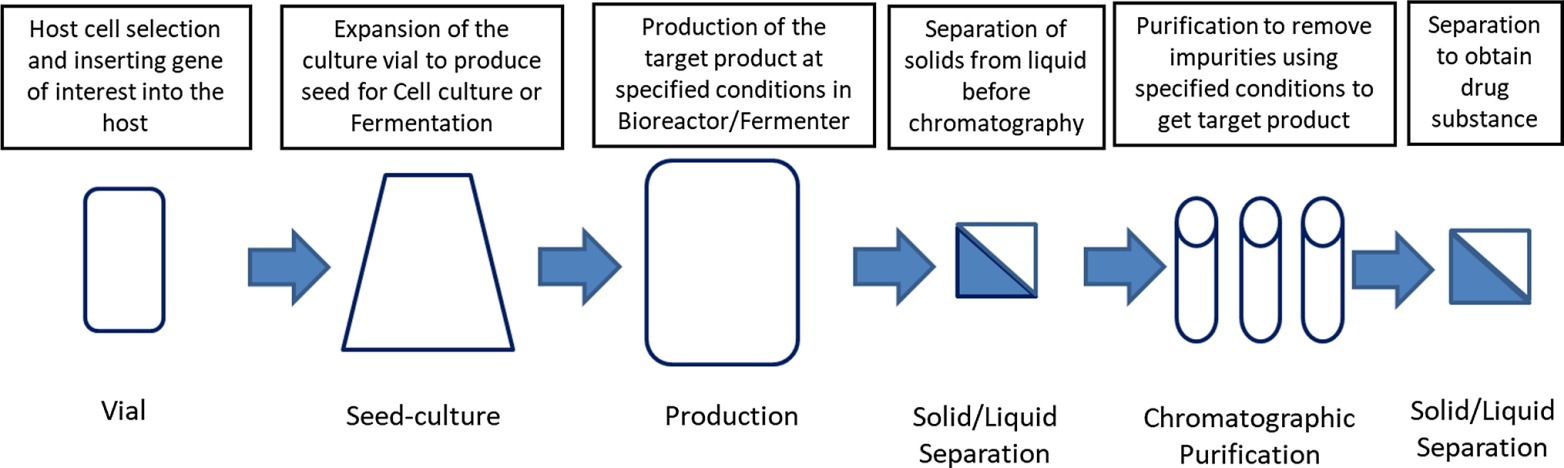
The process steps that could be involved in the manufacture of a biosimilar

In the process development and manufacturing of a biosimilar, the key characteristics of the originator molecule known as the CQAs need to be matched as closely as possible to the biosimilar product to ensure bio-similarity [58]. Biologics are highly complex molecules and, therefore, possess inherent variability from both the biological processes within the organisms used to produce them and the manufacturing process used for their production [58]. The proprietary nature of the manufacturing process of the reference product is another key challenge in the development of the manufacturing process for biosimilars [32]. The assessment of similarity between the innovator molecule and the biosimilar involves comprehensive structural and functional analysis [critical quality attributes (CQAs)] throughout the development process [32]. The availability of the analytical methods to analyze in-process samples for the CQAs during the process developmental stage is very essential in the development of biosimilars. An example of a manufacturing process is shown in Fig. 9.

The companies involved in the production of biosimilars range from small to mid-size and big biotech companies [35]. While some of the companies have the resources and capability to do everything from development to cGMP manufacturing in-house, other companies outsource some of the development and manufacturing to qualified contract research and manufacturing organizations (CROs, CMOs). In many respects, biosimilars are ideal products for developers to outsource development and commercial manufacturing to CROs and CMOs [34]. This is because CROs and CMOs have experience and expertise on working with many different systems and platforms which could be applied to the biosimilar product development, state-of-the art technology and the flexibility required in the development process [34]. Unlike a few decades ago where there were a few CROs and CMOs with the required expertise, a biosimilar developer today can find dozens of qualified CROs and CMOs and this can help shorten the timeline to complete product development [41].

Whether the product is being developed in-house by the biosimilar developer or being outsourced, a QMS for research and development will be needed to capture the development history of the product through archivable, retrievable and auditable systems. As previously described in the analytical portion of this paper, the QMS in process development should contain at least equipment calibration, document control and data integrity (Sect. 4). These include document management systems, learning management systems, equipment asset management and change management as described above (Sect. 2).

The evidence required for a biosimilar as opposed to originator molecule is to demonstrate similarity to the reference product with respect to quality, safety, and efficacy using a stepwise approach that includes analytical, nonclinical and clinical studies [32]. Although minor differences exist among US Food and Drug Administration (FDA), European Medicines Agency (EMA), Health Canada, World Health Organization (WHO), all require a stepwise approach that includes analytical studies, at least, one human pharmacokinetic (PK) and pharmacodynamic (PD) study, and generally a minimum of one efficacy and safety study intended to support a demonstration of biosimilarity [47]. To meet the FDA requirements, clinical development begins with comparable pharmacokinetics (PK), pharmacodynamics (PD) and immunogenicity with reference product in relevant population [2, 56]. Once PK, PD and immunogenicity similarity with reference product has been demonstrated, at least one Phase 3 clinical comparability trial is conducted to confirm similar efficacy and safety in a sensitive population [2, 52, 56].

Unlike innovator molecule, in the development of a biosimilar, it is very necessary to ensure that the biosimilar fits the constrained range for all CQAs of the originator molecule [58]. The target-directed development of a biosimilar can be done using quality by design (QbD) which is a systematic risk-based approach to the development of a product and associated manufacturing process. Using the fingerprint of the originator molecule, a set of CQAs whose functional and structural characteristics are relevant can be determined and used in the development of the biosimilar product [58]. During development, each of the steps in the process steps (a) cell line selection and engineering, (b) cell culture/fermentation development, (c) purification process optimization and (d) stable formulation is characterized for their impact on CQAs. This is achieved by analyzing the risk and performing experiments using design of experiments (DOE) or one factor at a time (OFAT) to generate process understanding of the impact of the process parameters on the CQAs. It is also important that the biosimilar is manufactured in a state-of-the-art cGMP facility that follows high-standard manufacturing guidelines and QbD principles [63]. Such a facility will ensure that rigorous in-process controls are implemented to monitor the biosimilar analytical fingerprint and reduce batch-to-batch variations [58].

## Conclusion

Biosimilars currently represent a small portion of the biopharmaceutical pipeline in the US and EU but is expected to expand due to many drugs coming off patents. The typically reduced timeline required for getting regulatory approval (from 12 to 8 years) and cost of development (10–20%) of innovator molecule is expected to be the driver for many companies. To further reduce the time for process development, QMS can help develop reliable lab systems and storage for archivable, retrievable and auditable data for efficient process development. The use of the deuterium-hydrogen exchange to probe biosimilar interactions gives a more thorough understanding of the structure–function relationship between the innovator and biosimilar molecule. The quality of the analytical packages can be strengthened with similarity data from the deuterium–hydrogen exchange in advance of clinical testing. Companies developing biosimilars may leverage the flexible CDMO model in development by taking advantage of the experience, knowledge and expertise available. The decision on process mode to be used such as batch, fed-batch, perfusion is based on time, cost, productivity and quality profile. A sample process was conducted at lab scale using single-use bioreactors and successfully scaled up to 40 L using P/V ratio. Each of the steps in the biosimilar process (a) cell line selection and engineering, (b) cell culture/fermentation development, (c) purification process optimization and (d) stable formulation needs to be characterized for their impact on CQAs. DOE (Design of experiment) or OFAT (one factor at a time) can be used to generate process understanding of the impact of the process parameters on the CQAs based on the risk assessment. It is critical to have a well-characterized molecule along with a robust control strategy and data package when filing with the regulatory agencies.
